# The effectiveness of local strains of *Fusarium oxysporium f. Sp. Strigae* to control *Striga hermonthica* on local maize in western Kenya

**DOI:** 10.1002/fsn3.1732

**Published:** 2020-07-09

**Authors:** Dorah A. Oula, John M. Nyongesah, George Odhiambo, Samuel Wagai

**Affiliations:** ^1^ School of Biological and Physical Sciences Maseno University Maseno Kenya; ^2^ School of Biological Sciences Rongo University College Rongo Kenya; ^3^ School of Biological and Physical Sciences Jaramogi Oginga Odinga University of Science and Technology Bondo Kenya

**Keywords:** biocontrol, *Fusarium oxysporium*, local maize, local strains, striga weed

## Abstract

*Striga hermonthica* weed infestation continues to persist among smallholder poor farmers in Kenya who depend on traditional maize (*Zea mays* L.) seeds for their livelihood. The purpose of this study was to evaluate the efficacy of five local *Fusarium oxysporum f. sp. Strigae* strains (FK1, FK2, FK3, FK4, and FK5) to control *Striga* on susceptible local maize cultivar “Rachar” in three farmer field sites in Siaya County, Kenya. A complete randomized block design was used in each site. Statistical analysis was done using SAS 9.1 software, and means for different strains were tested with Fisher's LSD. The strains differentially reduced the number of emerged *Striga* and infected most of the emerged *Striga*, which affected performance of the local maize. *Striga* emergence and infection rates were significantly different (*p* < .05) between different strains of *F. oxysporum f. sp. strigae*. FK1 and FK2 strains had the least pathogenicity, while FK5 strain had the highest pathogenicity on *Striga*. Soil and climatic factors influenced the rate of infection for the tested strains and maize performance. Improvement in yield during the short rains was attributed to the persistence of *Fusarium* strains in the soil. Based on *Striga* emergence and infection rates, and maize yield, FK5 was the most effective strain to curb *Striga* menace. Adoption of local *F. oxysporum* strains will increase maize yield in Siaya County's Striga‐infested fields from a dismal average of 0.95 t/ha to about 1.95 t/ha. The observed significant differences in the tested strains between sites for the infection and emergence rates revealed the importance of considering pathogens on a field‐to‐field basis. Further studies should be carried out to establish the relationship between soil properties and the five fungal strains.

## Graphical abstract

Study sites for biological control of witch weed.

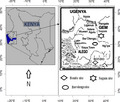

## 1 INTRODUCTION

Obligate parasitic weeds (*Striga hermonthica (del) Benth*) are major contributors to hunger, malnutrition, and food insecurity across sub‐Saharan by their effects on yields in major crops (Gressel et al., 2004; Schaub, Marley, Elzein, & Kroschel, 2006). These parasites attach onto the crop hosts’ roots before penetrating into the vascular system and eventually removing water, photosynthates, and minerals (Gressel et al., 2004; Joel, 2000; Yonli et al., 2005). Since crop yield is reduced when crops are infested with *Striga,* there is need for preventing the production of new seeds and increasing the crop yield in *Striga‐*infested land through feasible on‐farm management solutions. Parasitic *Striga* weed is a major biotic constraint to increased cereal production for millions of rural farm families in sub‐Saharan Africa (Atera, Ishii, Onyango, Itoh, & Azuma, 2013; Khan, Midega, Amudavi, Hassanali, & Pickett, 2008).

Although the use of a multiple integrated management approach for controlling *Striga* infestations has been commonly proposed (Parker & Riches, 1993), *Striga* has remained noxious and difficult to control (Ali‐Olubandwa, Kathuri, Wanga, & Shivoga, 2011). Several control measures that involve resistant host crop varieties, chemicals, crop rotation, intercropping with *Striga* host and nonhost crops, and soil fertility management have been applied in Africa on various crops (Chitere & Omolo, 1993; Kanampiu et al., 2003; Khan et al., 2008). However, the success of most of the available approaches to control *Striga* may be limited due to biological and socio‐economic reasons (Khan et al., 2008; Oswald, 2005). Furthermore, local knowledge may be relevant to the rural marginalized population but the high costs of synthetic pesticides and associated toxicity risks may discourage their integration in pest management systems (Khan et al., 2008).

Although researchers in Africa have intensified studies on *Striga* control (Atera et al., 2013; Kabambe, Kauwa, & Nambuzi, 2008; Kanampiu et al., 2013; Oswald, 2005), more efforts are needed to develop cost‐effective and environmentally friendly control options for the poor local farmers.

Traditionally, many farmers have relied on agronomic practices such as hand pulling, soil fertility improvement, herbicides/chemical control, use of trap crops and intercropping with legumes, and use of *Striga*‐tolerant crop varieties to manage *Striga* weed (Achola, 1999; Khan et al., 2008; Olakojo & Olaoye, 2005; Woomer et al., 2004). In Kenya, these methods have been costly, labor‐intensive, and beyond the means of local farmers (Olakojo & Olaoye, 2005). For instance, hand pulling is labor‐intensive, soil fertility and chemical control are both expensive to local farmers and contaminate the environment, rotational cropping requires several years before the *Striga* seed bank can be reduced, and use of trap crop or intercropping may be expensive for resource‐poor farmers who can only afford single crop per cropping season. Additionally, the continual cultivation of susceptible cereal crops in Siaya County has led to the production of more *Striga* seeds and hence unintentional contamination of crop fields. Since most farmers depend on the cropping system where high frequency of cereals is combined with limited legume rotation and low use of fertilizer in Siaya County, an alternative approach of using local strains of *Fusarium oxysporum f. sp. strigae* may alleviate *Striga* situation.

The potential for biological control of *Striga* weed has received enormous attention in the recent past (Joel, 2000; Kanampiu et al., 2013; Nzioki et al., 2016; Olakojo & Olaoye, 2005; Schaub et al., 2006; Shayanowako, Hussein, Laing, & Mwadzingeni, 2020; Yonli et al., 2005; Zarafi, Elzein, Abdulkadir, Beed, & Akinola, 2015), with most studies focusing on soil microorganisms of the genus *Fusarium* (Fungi of genus *Fusarium*) as an option to biologically control its infestation in cereal crops. (Ciotola, Hallett, & Watson, 1995; Mrema, Shimelis, Laing, & Bucheyeki, 2017; Nzioki et al., 2016; Schaub et al., 2006; Yonli et al., 2005). Various fungi have been tested for pathogenicity on *Striga* with *Fusarium* species as the most prevalent fungi associated with diseased *Striga* plants (Ciotola et al., 1995). Currently, *Fusarium spp* have been isolated from diseased *Striga* plants with surmountable success (Ciotola et al., 1995; Jumjunidang & Soemargono, 2012).

*Fusarium* spp. are long‐lived soil inhabitant that can survive extended periods in the absence of their host by colonizing crop debris and producing chlamydospores, dormant resting propagule. Since *Striga* growth interacts with local conditions (Oswald, 2005), any approach must strive to integrate *Striga* biologically for designing an efficient control method. *Fusarium* spp. can be persistent in the soil as saprophytes or can invade root epidermal and cortical tissues either pathogenically or nonpathogenically (Yonli et al., 2005). However, exigent questions remain unanswered regarding the activity of local *F. oxysporum* strains in controlling *Striga* weed in maize. The major threat to livelihoods of smallholders persists in Siaya County (western Kenya) due to *Striga* weed by impacting negatively on maize yield. Agriculture and fishing are the main economic activities in this county. Local farming systems are characterized by a very small landholding size with very low external input use and declining soil fertility (Place, Adato, & Hebinck, 2007). Since biocontrol agents have the potential to protect seeds as well as colonize the rhizosphere when added as seed treatments, and may protect the subterranean portions of growing plants from attack by plant pathogens (Ahmad & Baker, 1987), we hypothesized that biocontrol agent saprophytically colonizes the roots of the maize seedlings differentially in different agroecoregions reclaiming plant as it establishes. The main objective of this study was to evaluate the effects of *Striga* weed on local maize performance and assess the effects of inoculation of maize seeds with local strains of *Fusarium oxysporum f. sp. Strigae* in different agroecological regions.

## 2 MATERIALS AND METHODS

### 2.1 Study site

**Figure 1:**
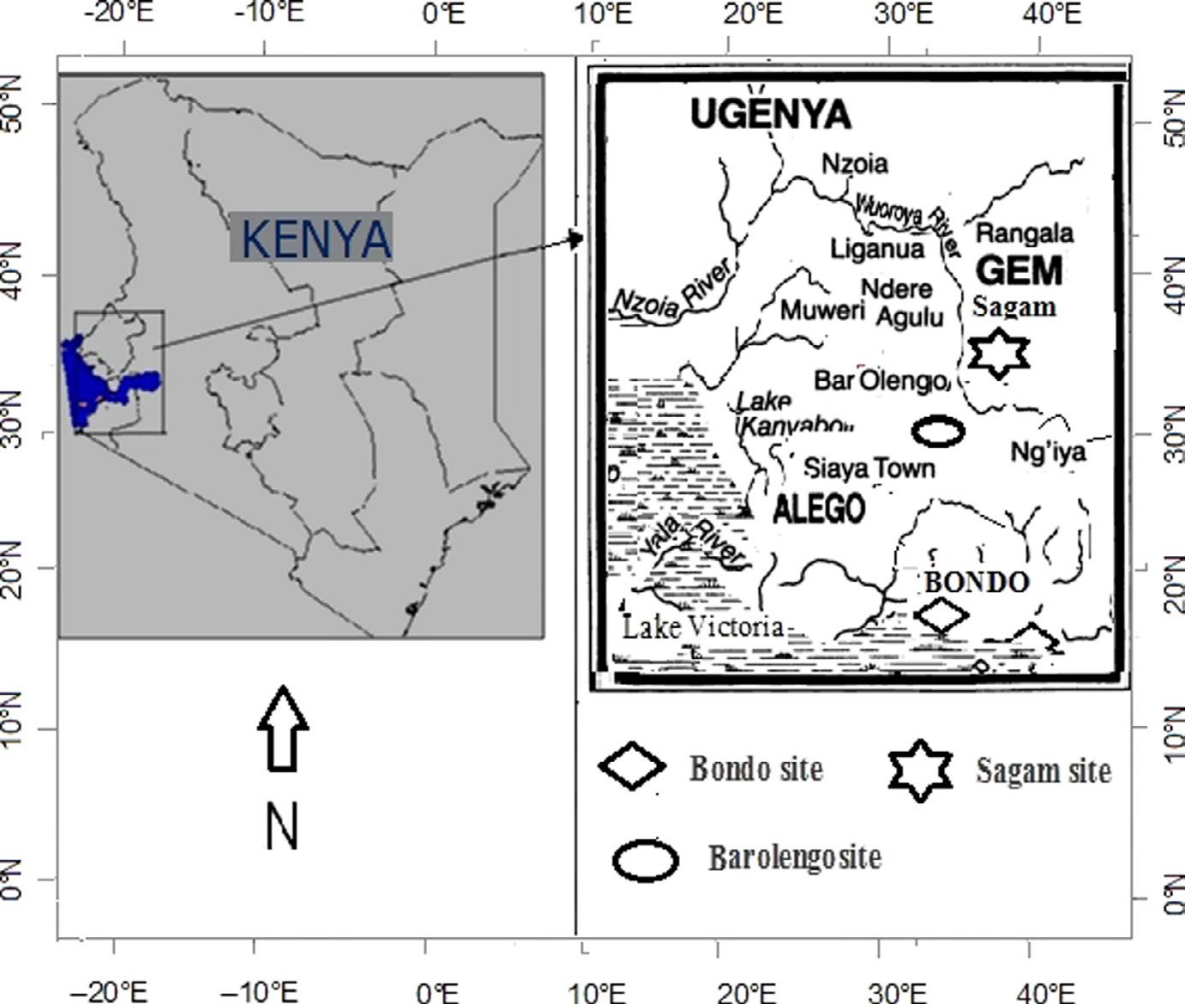
Location of study site in western Kenya

**Figure 2:**
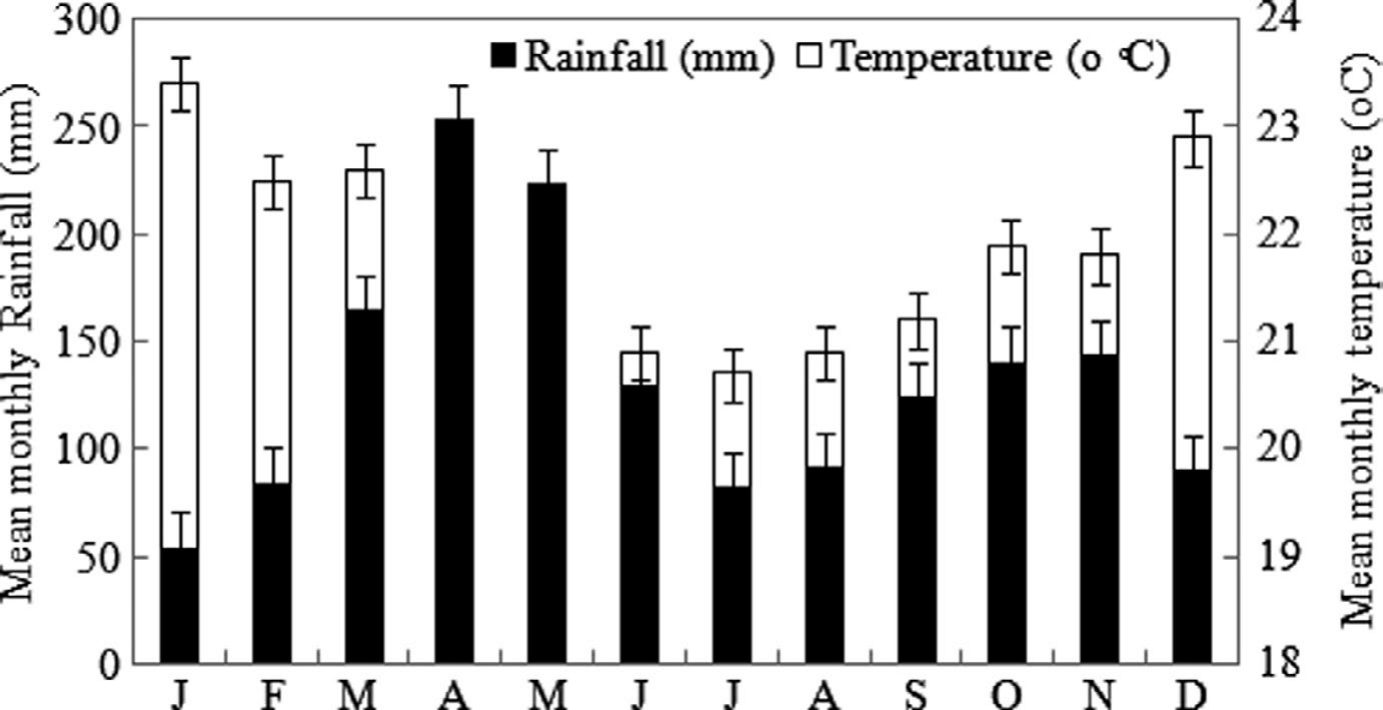
Rainfall and temperature of Siaya County

This study was carried out at three farmers’ fields with history of *Striga* infestation in Bondo (0°25′ to 0°2′ S, 34°0′ to 34°33′ E), Sagam (0°26′ to 0° 18′ S, 33° 58′ to 34° 33′ E), and Barolengo (0°1′0″ N and 34°12′0″ E), in Siaya County, Kenya (Figure 1). Siaya County received a bimodal rainfall pattern with long rains starting from mid‐March to June with the peak in April and May. The short rains started in September, peaked in October, and ended in November. The average annual rainfall was 1,577 mm, while the annual mean temperature was 21°C where January was the hottest month (Figure 2). These values were consistent with previous findings by Kiplangat, Omenyo, Njoroge, Okalebo, and Ahoya (2013). Agriculture and fishing are the main economic activities in the county where local farming systems are characterized by a very small landholding size with very low external input use, declining soil fertility, and exodus of able‐bodied men to secure jobs in urban areas (Place et al., 2007). Based on soil sampling that was carried out during this study, the soils of this county were deep and friable, but some places were shallow lying over murram layer. Sagam site had valley swampy black soil, which was classified as clayey black cotton soil. Barolengo had Ferralsols, which are common soils in South Nyang'oma and Usigu divisions. Bondo site had poor shallow soils of sandy loams and Acrisols. The three sites recorded a pH of 4.1, 6.4, and 4.0 for Bondo, Sagam, and Barolengo, respectively (Table 1). The pH values for Bondo and Barolengo sites were way below the required minimum (5.5) for maize growth and development (Kiplangat et al., 2013). Generally, Sagam site recorded higher soil nutrients compared to Bondo and Barolengo. Total N of 0.35%, 0.17%, and 0.15% was recorded at Sagam, Barolengo, and Bondo, respectively. Organic carbon was also higher at Sagam (1.49%), while Bondo had low values (0.83%).

**TABLE 1:** Soil properties of three study sites in Siaya County

Site	Soil type	CEC (%)	PH	Total *N* (%)	Total P (mg/100 g)	Organic C (%)
Bond	Acrosol	68.9 (3)	4.1 (3)	0.15 (5)	17.6 (5)	0.83 (5)
Sagam	Black cotton	95.3 (3)	6.4 (3)	0.35 (5)	13.4 (5)	1.49 (5)
Barolengo	Ferralsols	38.2 (3)	4.0 (3)	0.17 (5)	23.4 (5)	1.12 (5)

Note

Values in bracket represent sample size.

### 2.2 Experimental design

The *Fusarium oxysporium f. sp. Strigae* strains from five different ecological sites were used to inoculate maize seeds before planting. A randomized complete block design was used in this study where three blocks were set up in each site. The land measured 28 × 25 m in each site. A total of 18 plots per site measuring 5.5 × 3.75 m were established. The plots were arranged in 3 blocks (replicates), with each block consisting of 6 plots. A parallel control was maintained during the growing period where seeds were planted without inoculant. The experiment was carried out during the long rains and short rains of 2013. Effectiveness of *F. oxysporum f. sp. Strigae* strains to control *Striga* was evaluated in susceptible cultivar of maize “Rachar.” This was carried out in 3 sites using 5 sources of isolate plus 1 control. The sources were as follows: FK1 from Kosele center, FK2 from Kosele farm, FK3 from Siaya ATC (agricultural training center), FK4 from Bondo, and FK5 from KARI‐CIMMYT, Kibos.

### 2.3 Isolation of fungal strains and molecular characterization of the isolated fungi

Samples of diseased *S.hermonthica* plants showing signs of necrosis and wilting were uprooted from farms in Kosele in Rachuonyo subcounty; KARI‐CIMMYT, Kibos; Siaya ATC; and farms in Bondo subcounty. They were placed in paper bags before transportation to the Maseno University Laboratory. The *Striga* plant samples were cut into small pieces up to 1.5 cm length and placed in a suitable sterilizing solution (1% sodium hypochlorite, NaOCl) for 1–5 min. The tissues were removed from sterilizing solution and then rinsed thoroughly (3 times) with distilled water. Half‐strength potato dextrose agar (PDA) was prepared and amended with chloramphenicol antibiotic, then incubated at 25°C for 18–24 hr, and observed to check out for a pinkish/purplish mycelia establishment. Series of dilutions were prepared in sterile water using a plug of the grown characteristic fungal mycelia. Observations were made using compound microscope to guide the plating dilution(s) and plating out of 0.1 ml for establishment of pure/single colonies from several dilutions and plate on ½ PDA sterile media. The more the dilutions, the fewer the colonies established, and the distinctive/pure the culture became thus easy isolation. Procedure for repeat plating for more pure cultures was carried out until a clean culture (pinkish/purplish) was obtained.

Molecular characterization of the isolated fungi was done to confirm their morphological identities. DNA was extracted from fungal isolates grown on PDA plates for 7 days. Their mycelia were harvested and resuspended in nuclease‐free water. Total DNA was extracted from the resuspended mycelia of each isolate (50–100 mg wet weight) using a ZR Fungal/Bacterial DNA MiniPrep Kit (Zymo Research, South Africa) according to the manufacturer's instructions. The obtained DNA sequences were blasted using the NCBI BLAST (National Center for Biotechnology Information—Basic Local Alignment Search Tool) to reveal their identities. To reveal the relatedness of the isolates, alignment of all the sequences was done using Clustal X 2.1 software and phylogenetic analyses were conducted using MEGA version 5.1 using the neighbor‐joining (NJ) method (Tamura *et al.,* 2011). In the NJ analysis, distances were calculated using the Kimura 2‐parameter model and bootstrap tests performed with 1,000 replications. The obtained phylogenic tree is presented as supplementary data.

### 2.4 Seed dressing and field management

Local maize seeds of “Rachar” were primed for 10 hr and dressed at the rate of 40 g of *F. oxysporum* per 600 seeds at Maseno University microbiological laboratory based on Woomer et al. (2004). The control seeds were dried without any dressing. All the seeds were placed under shade to dry for both long rain and short rain seasons.

*Striga hermonthica* seeds were obtained from the KARI‐CIMMYT collaborative facilities at Kibos.

These seeds had been collected from fields in Siaya County that were heavily infested by *S. hermonthica*, that is, >10 Striga shoots m^−2^. The seed was already formulated into a *S. hermonthica*:sand mixture of 1:4 as described by Avedi et al. (2014). One tablespoonful of *S. hermonthica* seed–sand mixture (1,000 *S. hermonthica* seeds) was placed in every planting hole. The seeds of maize were sown in ridges with a spacing of 70 cm between rows and 30 cm within rows in each site. Two maize seeds were planted in each hole where rows were arranged horizontally in each site. There were a total of 9 rows having 8 plants in a row, making a plant population of 72 per plot. Fertilizer (DAP granules) was applied at the rate of 1 teaspoonful per planting hole. Two weeks after germination, the seedlings were thinned to one per hole. Hand weeding was done after every two weeks for all weeds except *S. hermonthica*. The weeds were removed thereafter continually by hand pulling to avoid interactions with *Striga* development.

### 2.5 Data management and analysis

#### 2.5.1 Striga incidence

The number of emerged *S. hermonthica* plants within 15 cm radius of each maize stem was recorded. The proportion of *S. hermonthica* plants expressing wilt symptoms was used to calculate percentage infection rates. Severity of wilting in *S. hermonthica* was estimated using a visual rating scale based on modified version by Olakojo and Olaoye (2005).

#### 2.5.2 Maize performance

At maturity, the number of maize cobs on the sampled plants per plot was counted. The total weight of maize cobs per plot was measured using a portable electronic scale (Constant 14192‐7, South Korea). Grain moisture content was determined by randomly picking 3 cobs per plot, then extracting grain from each cob, and placing the three in a grain moisture meter (GMK‐303A, G‐WON HITECH Co., Ltd, Seoul, South Korea). This was done in triplicate. The weight of the grains per plot and the moisture content (MC) were used to determine yields in tonnes per hectare using the formula described by De Groote (2002) as shown below:

$$\text{Yield}\;\left({\text{ton ha}}^{-1}\right)=\frac{\text{FW}\;\left(100-{\text{MC}}_{1}\right)\times S\times 1000\;m^{2}}{\left(100-{\text{MC}}_{2}\right)\times {\text{Pm}}^{2}\times 1000\;\text{kg}}$$

where *FW* was weight of harvested cobs (Kg); *MC1* was the moisture content (%) in grains at harvest; *MC2* was the required moisture in maize grain at storage (i.e., 13%); S was the shelling percentage (85%); and P = plot size.

All data were subjected to analysis of variance (ANOVA) with means per plot combined across the season for *Striga*‐infected and *Striga*‐free conditions using Statistical Analysis System (SAS) software, version 20 (SAS Institute Inc., 1990). Where the *F* test was significant at *p* ˂ .05, strains and site differences were tested by least significant difference (LSD) at 5% level of probability.

## 3 RESULTS

### 3.1 *Striga* emergence and infection rates

Bondo site had the highest Striga emergence rates (%) followed by Barolengo and Sagam with the least rates of emergence. All the tested Fusarium strains performed differently within and between sites during the long and short rain seasons. Sagam and Barolengo sites recorded lowest *Striga* emergence in the FK5 strain plots compared to other treatment plots throughout the growing season with maximum rates of 4.8 ± 0.8 and 5.8% ± 1.1%, respectively (Table 2), during the long rains. Among the tested strains, different trends were observed in terms of Striga emergence rates, that is, Bondo site FK1 > FK2 > FK4 > FK3 > FK5, Sagam site FK4 > FK2 > FK1 > FK3 > FK5, and Barolengo site FK2 > FK1 > FK4 > FK3 > FK5 during the long rains. Highest emergence was observed at Bondo (53 ± 5.1%) followed by Barolengo (46.6 ± 8) and Sagam (43.8 ± 4.1) in the control plots.

**TABLE 2:** Mean *Striga* Emergence rates (%) during the growing seasons

Long rain season				Short rain season		
Treatments	Sites			Sites		
	Bondo	Sagam	Barolengo	Bondo	Sagam	Barolengo
FK1	23.4a	11.2a	16.6a	11a	15.3a	17a
FK2	14.8b	11.4a	22.2b	12.6a	12.3b	9.7a
FK3	11.2c	8.2b	6.6c	5.7a	3.7c	5.3a
FK4	14.4b	12a	11.2d	11a	6.7d	11a
FK5	8.6c	4.8c	5.8c	6a	5.7d	6.7a
C	53d	43.8d	46.6e	31.7b	25.7e	24.7a
*p* value	.00007	.00002	.00003	.002	.005	.49
LSD (0.05)	2.3	2.3	3.2	7.4	2.8	NS
%CV	7.8	9.4	8.4	7.4	7.1	5.8

Note

Means followed by the same letters in the same column are not significant.

Abbreviations: CV, coefficient of variance; NS, not significant.

Similar to long rains, the control plots recorded the highest Striga emergence rates where Bondo (31.7% ± 4.1%), Sagam (25.7% ± 6.1%), and Barolengo (24.7% ± 12.6%). These values were slightly lower compared to the long rains. On the basis of the tested fungal strains, the highest Striga emergence rates were recorded at Barolengo in FK1 strain (17% ± 11.5%). Sagam site had lower emergence rates followed by Barolengo and highest rates at Bondo during the short rain season. Based on strains’ performance during the short rain season, Bondo FK2 > FK1 > FK4 > FK3 > FK5, Sagam FK1 > FK2 > FK4 > FK5 > FK3, and Barolengo FK1 > FK4 > FK2 > FK5 > FK3 striga emergence rates. Therefore, FK3 and FK5 strains recorded the least Striga emergence rates in all the three sites and during the two seasons. Based on Striga emergence rates, all the tested strains were significantly different (*p* < .05) in all sites except Barolengo during the short rain season.

The rates of *Striga* infection by *F. oxysporum f. sp. strigae* varied between the three sites and seasons. The control plot recorded the lowest infection rates in all sites where Bondo had no infection during the long rains (Table 3). Sagam and Barolengo sites recorded 1.5% ± 1.1% and 0.8% ± 0.81%, respectively, in the control plots during the long rains. Highest rates of Striga infection recorded in FK3 and FK5 strains at Sagam site were 59.4% ± 5.7% and 77.4% ± 11.8% during the long and short rain, respectively. Apart from Bondo site, FK3 and FK5 strains had >50% infection rates throughout the growing seasons. FK1 and FK2 strains recorded <40% infection rates in all sites. Significant differences in Striga infection rates were observed between strains in all sites during the study period. All the tested strains were significantly different (*p* < .05) in all sites and seasons. However, no significant differences were observed between FK1, FK2, FK3, and FK5 strains at Bondo site during the long rains.

**TABLE 3:** Mean *Striga* Infection rates (%) by *Fusarium oxysporum f. sp. Striga* strains

Long rain season				Short rain season		
Treatments	Sites			Sites		
	Bondo	Sagam	Barolengo	Bondo	Sagam	Barolengo
FK1	32.81a	29.5a	25.4a	34.8a	21.2a	31.1a
FK2	33.8a	37.4b	12.7b	36.1a	33.1a	21.4b
FK3	42.7a	53.6c	52.7c	53.3b	61.3b	53.9c
FK4	25b	18.5d	14.2b	42.7c	25.8a	41.9d
FK5	45.2a	59.4e	57.4d	53.7b	77.4c	59.0c
C	0c	1.5f	0.8e	1.7d	7.6d	3.4e
Mean	29.9	33.4	27.2	37.1	37.7	35.1
*p* value	.01	.00002	.00003	.001	.00009	.003
LSD (0.05)	14.3	4.6	3.5	3.77	11.9	8.4
%CV	5.5	6.5	8.4	5.2	6.9	5.9

Note

Means followed by the same letters in the same column are not significant.

Abbreviations: CV, coefficient of variance; NS, not significant.

### 3.2 Grain yield

The highest yield was exhibited by FK5 strain during the long rains and FK3 during the short rain season. On average, Sagam site recorded the highest yield compared to Bondo and Barolengo in both seasons (Table 4). Maximum recorded yield during the long rain and short rain seasons was 1.95 ± 0.15 t/ha and 1.93 ± 0.01 t/ha, respectively. At Sagam site, the control plot had consistently lower yield compared to all the tested strains, while some strains at Bondo and Barolengo had lower yield compared to the control. There was an improvement in yield during the short rain season in the strains at Bondo and Barolengo sites, and three strains except FK4 and FK5 strains at Sagam site. Apart from Sagam site during the short rain season, there were no significant differences (*p* < .05) between all the strains in all sites. At Sagam site, significantly differences (*p* < .05) were recorded for FK1, FK3, FK4, and the control. However, no differences were recorded between FK1 and FK2, FK3, and FK5.

**TABLE 4:** Maize yield (t/ha) at the end of growing seasons

Long rain season				Short rain season		
Treatments	Sites			Sites		
	Bondo	Sagam	Barolengo	Bondo	Sagam	Barolengo
FK1	1.23 ± 0.03a	1.54 ± 0.1a	0.99 ± 0.05a	1.48 ± 0.10a	1.71 ± 0.07a	1.39 ± 0.16a
FK2	1.20 ± 0.05a	1.57 ± 0.04a	0.93 ± 0.04a	1.45 ± 0.11a	1.71 ± 0.0a4	1.43 ± 0.12a
FK3	1.26 ± 0.04a	1.69 ± 0.04a	1.29 ± 0.03a	1.29 ± 0.05a	1.93 ± 0.01b	1.62 ± 0.07a
FK4	1.13 ± 0.02a	1.82 ± 0.11a	1.12 ± 0.03a	1.46 ± 0.02a	1.80 ± 0.12c	1.32 ± 0.05a
FK5	1.27 ± 0.04a	1.95 ± 0.15a	1.37 ± 0.34a	1.49 ± 0.13a	1.92 ± 0.04b	1.55 ± 0.04a
C	1.22 ± 0.01a	1.52 ± 0.21a	0.95 ± 0.03a	1.19 ± 0.09a	1.36 ± 0.16d	1.34 ± 0.12a
*p* value	0.2	0.1	0.2	0.2	0.008	0.3
LSD (0.05)	NS	NS	NS	NS	0.06	NS
%CV	5	11.4	13.4	10.5	7.4	11.1

Note

Values represent mean yield ± *SE*. Means followed by the same letters in the same column are not significant.

Abbreviations: NS, not significant.

## 4 DISCUSSION

All the *F. oxysporium* strains had significantly higher rates of infection compared to the control. Since the strains were obtained from different sources, many factors may affect their efficacy in controlling *Striga* weeds. The differences in their efficacy suggest that similar phylogenetic groups of *F. oxysporium* may not be identical and hence have little connection in controlling *Striga* weed since a range of factors influences the rate of infection (Atera et al., 2013). Erratic rainfall and high temperature experienced in Siaya during the study period may have contributed to high *Striga* infection rates and hence low rates of emerged *Striga* in the three sites. Irregular rainfall during the growing season led to low moisture content that reduced germination rates and favored the recorded *F. oxysporum f. sp. strigae* infection rate. Although we did not monitor soil moisture content during the study period, the importance of soil moisture on pathogen development has been well documented in previous studies (Doohan, Brennan, & Cooke, 2003; Kanampiu et al., 2003). This assertion may be valid since variability in climatic conditions such as rainfall has been shown to affect emergence of Striga plants (Yonli et al., 2005).

Reduced efficacy of *F. oxysporum* f. sp. Strigae in both seasons at Bondo site was attributed to the low pH compared to Sagam and Barolengo sites. Saline soil produces a poor soil–water–air relationship, which may result in a poor plant growth and deficiency of iron or other micronutrients (Woomer et al., 2004). This was not surprising since approximately 1 million hectares of land in western Kenya is acidic with a pH of less than 5.5 and hence the consequences of P deficiencies (Achola, 1999; Woomer et al., 2004). Therefore, the presence of acidic soils in these sites may have affected the proliferation of the fungal strains in the soil and led to poor physical soil properties, which resulted in a reduced *Striga* control. Furthermore, the obtained pH values for Bondo and Barolengo sites were way below the required minimum (5.5) for maize growth and development (Kiplangat et al., 2013). Since stunted growth is associated with acidity (Okalebo et al., 2005), the observed low height in maize at Barolengo may have been driven by this phenomena.

The observed low emergence and high infection rates in the local *F. oxysporum f. sp. Strigae* strains may be attributed to destruction of *Striga* seeds and wilting of emerged plantlets. Such results have been observed in previous studies where Striga population reduced due to application of *F. oxysporum f. sp. strigae* isolates (Atera et al., 2013; Avedi et al., 2014; Ciotola et al., 1995; Shayanowako et al., 2020; Yonli et al., 2005). On average, the efficacy of *F. oxysporum* f. sp. Strigae in controlling Striga was relatively low in Siaya County (i.e., 53%—long rains and 63%—short rains) compared to foreign isolates carried out in west Africa where 75%–90% were recorded (Abbasher, Hess, & Sauerborn, 1998). Based on each field, Sagam site recorded efficacy of 77% during the short rain season which was better than *F. oxysporum* f. sp. strigae obtained from North Ghana and tested in western Kenya by Avedi et al. (2014). The relatively low Striga infection rates may have been caused by our experimental protocol where isolation was carried out from *Striga*‐infected plants but no isolation was done from the soil. Since previous studies have indicated that soil‐inherent pathogens can suppress the effects of fungi on *Striga* weed (Abbasher et al., 1998), it is likely that these played a major role in low rates and hence future studies should involve soil *Striga* isolation. Additionally, soil sampling for microflora analysis to assess the indigenous microflora should be assessed in future studies for their contribution in controlling *Striga* emergence.

Several studies have indicated *S*. *hermonthica* has devastating effects on grain yield of susceptible maize by robbing its host of carbon, nitrogen, and inorganic salts (Kabambe et al., 2008; Kanampiu et al., 2003). This diminishes the growth and photosynthetic capacity of cereal crops (Khan et al., 2008). Yields on the three farmer's fields were generally low. Based on highest performing strains, maximum yield during the long rains (1.27, 1.95, and 1.37 t/ha) and short rains (1.49, 1.92, and 1.62 t/ha) was obtained at Bondo, Sagam, and Barolengo sites, respectively. These were lower values compared to world average of 4.2 t/ha and expected yields obtained at research centers of 7–8 t/ha (Okalebo et al., 2005). However, these values were slightly higher compared to average maize yields in Siaya County farms where farmer planted their maize without fertilizer (below 0.5 t/ha). Although information on maize yield under fertilizer application in Siaya County is scarce, improved yield to a maximum of 1.5 t/ha in the three sites within the control plot in this study could have resulted from the combined influence of fungal isolates and inorganic fertilizer that was applied at sowing.

Remarkable high yield was observed at Sagam site in all the tested *Fusarium* strains compared to Bondo and Barolengo sites. Since Bondo and Barolengo sites had low pH, soil micronutrients were unavailable to plants and hence may have affected their growth vigor. Additionally, precipitation received during the study period was comparatively low and could have contributed to the recorded yield in all sites. On average basis, significantly higher yield was observed in FK5 strain compared to other *Fusarium* strains during the long rains while FK3 and FK5 strains yielded higher during the short rain season. Observed differences in yield conferred by activity of different strains of *F. oxysporum* f. sp. strigae may be due to differences in biotic and abiotic factors.

These findings confirm previous studies on other fungal strains that have indicated that inhibitive endophytic and rhizosphere microbes are among biotic factors linked to reduced performance of *F. oxysporum* and other microbial biological control agents (Avedi et al., 2014). Although it is not clear why all the five *F. oxysporum f. sp. strigae* strains performed differently within the same site and between the three sites, improvement in yield in all sites during the short rain was a result of persistent soil protection conferred by the presence of the fungal strain in the soil and reduction of striga seedling in the soil. Improvement in maize yield in treatment plots may have been caused by mycorrhization between maize plants and *Fusarium* strains, hence proving the pathogens’ ability of improving crop yield as well as preventing further *Striga* distribution and infestation in the three farm fields. Additionally, high nutrient levels observed in the soil at Sagam site may have contributed to the recorded high yield in both seasons.

Lack of remarkable improvement in maize yield at Bondo site may have been caused by the high rates of *Striga* infestation at the site evidenced from high rate of *Striga* emergence. Additionally, the soils in Bondo and Barolengo were Acrisols and Ferralsols, respectively, which may have contributed to the low yield and the observed lack of consistency among the tested *Fusarium* strains at Bondo site. Additionally, improvement of soil physical characteristics conferred by the pathogen through ameliorating deficiencies of soil nutrients may partially be responsible for yield differences in all the three sites. This was partly reasonable since available P, N, and C were very low in all the study sites. Deficiency of these nutrients may have been the dominant factor that led to lack of vivid differences among five strains and the control plot at Bondo and Barolengo sites. However, future studies should be carried out to establish the relationship between soil properties and the five fungal strains.

## 5 CONCLUSION

The tested fungal strains showed high level of efficacy on the weed revealing a wide variety of local choices in developing a biological control against the weed. Three fungal strains (FK3, FK4, and FK5) demonstrated infection rates greater than 40%, indicating that the strains tested could easily colonize the weed. However, it was only the two strains of *Fusarium oxysporum* (FK3 and FK5) which had infection rates <50% in all the three sites. This demonstrated the suitability of *F. oxysporum* as a biocontrol agent against the weed. The less than 40% mortality rate exhibited by the two strains (FK1 and FK2) can be attributed to differences in abiotic factors within the study sites. Based on high grain yield and reduced *Striga* count per plot in each of the three sites, FK3 and FK5 were the most appropriate *F. oxysporum* strains for adoption by farmers in Siaya County. Adoption of local *F. oxysporum* strains will increase maize yield in Siaya County's Striga‐infested fields from a dismal average of 0.95 t/ha to about 1.95 t/ha. It is also expected that the use of local *F. oxysporum* strains will lead to reduction in Striga seed bank in the soil as observed in improved yield in subsequent growing season, hence greatly reducing the threat posed by the weed to maize farming and farmers’ livelihoods. Since all the tested *F. oxysporum* strains performed differently between the three regions, application of local *F. oxysporum* strains must be considered on a field‐to‐field basis. Considering the observed differences in maize performance between the two seasons and different strains in all the sites, future studies should consider continuous monitoring of the effects of environmental factors such as soil moisture content and temperature on local strains. Furthermore, the puzzle that needs to be resolved is why *F. oxysporum* f. sp. strigae isolates performed differently within the same site and between the three sites.

## CONFLICT OF INTEREST

The authors declare no conflict of interest.

## ETHICAL APPROVAL

This study conforms to the Declaration of Helsinki, US, and/or European Medicines Agency Guidelines for human subjects. The study protocols and procedures obtained were ethically reviewed and approved by Maseno University Ethical Review Committee. Informed consent was obtained from farmers who surrendered their land for research. Animal and human testing was not necessary in this study.

## Supporting information


**Data S1.** Based on the tree from the phylogenic tree, it is evident that the Kenyan isolates FK1 to FK5 (Foxy Kenya) were genetically identical and belonged to a single Clade (Clade 1). This Clade constitutes fungal isolates identified as Fusarium oxysporium at 97% bootstrap support values in comparison to Genbank isolates.
