# Supplementary material for: The effectiveness of local strains of Fusarium oxysporium f. Sp. Strigae to control Striga hermonthica on local maize in western Kenya
Source: Food Sci Nutr. 2020 Jul 9;8(8):4352–60. doi: 10.1002/fsn3.1732 (PMC7455961; doi:10.1002/fsn3.1732)
Supplement: Supplementary file 1 — Data S1. Based on the tree from the phylogenic tree, it is evident that the Kenyan isolates FK1 to FK5 (Foxy Kenya) were genetically identical and belonged to a single Clade (Clade 1). This Clade constitutes fungal isolates identified as Fusarium oxysporium at 97% bootstrap support values in comparison to Genbank isolates. [file FSN3-8-4352-s001.doc]

## Fusarium oxysporum Phylogenic tree


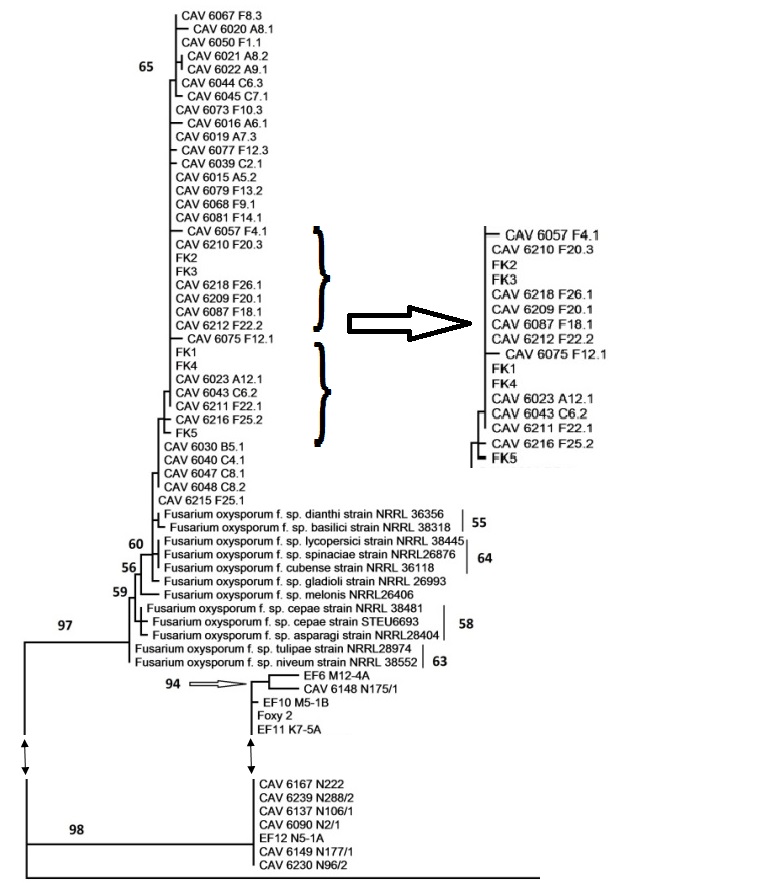


*The isolates were then identified as described earlier in section 23, by means of molecular and morphological techniques. These were the Fusarium species found to be associated with Striga, of which F. oxy appears to be the most predominant species. The Kenyan isolates belongs to a single linage, as none of them were found to pair with any isolates of the other 2 groups. For the phylogenetic studies, only foxy isolates were used to draw up a maximum parsimony phylogenetic tree, using the Translocation Elongation Factor α-1 (TEF) gene region. The foxy isolate collection were compared to various other foxy f. sp strains and the Kenyan isolates grouped together and formed a distinct clade. Based on the tree from the phylogenic tree, it is evident that the Kenyan isolates FK1 to FK5 were genetically identical and belonged to a single Clade (Clade 1). This Clade constitutes fungal isolates identified as Fusarium oxysporium at 97% bootstrap support values in comparison to Genbank isolates. Slight intraspecific variations of some strains correlate well with phylogenetic species concepts, and do not indicate that they were two clades.*
